# The impact of long-term prescription policy on primary care utilisation and costs among hypertensive patients in China: a six-year longitudinal study

**DOI:** 10.7189/jogh.15.04021

**Published:** 2025-01-17

**Authors:** Chunlu Yu, Lei Zhang, Luying Zhang, Wen Chen

**Affiliations:** 1 School of Public Health, Fudan University, Shanghai, China; 2 Shanghai Ninth People’s Hospital, Shanghai Jiao Tong University School of Medicine, Shanghai, China

## Abstract

**Background:**

China has recently implemented a long-term prescription policy as a component of the family doctor system in order to strengthen chronic disease management. In this study, we evaluated the net policy impact on health care utilisation and costs at community health centres (CHCs) among hypertensive patients.

**Methods:**

The study population included 164 857 hypertensive patients from a provincial capital city in Eastern China, with an average age of 69.93 years in 2014. We collected their health care utilisation and costs from 1 January 2014 to 31 December 2019 from the medical insurance claims database. The long-term prescription policy, implemented in 2018, allows patients registered with family doctors to obtain up to three-month prescriptions. We applied the multi-stage difference-in-differences model to examine the policy’s impact, comparing health care utilisation and costs between those eligible and for the long-term prescription policy and those who are not.

**Results:**

The long-term prescription policy significantly reduced hypertensive patients’ annual outpatient visits by 2.47 at CHCs and 0.18 at pharmacies, as well as prolonged the interval of prescriptions by 3.10 days at CHCs. It decreased the annual drug costs at pharmacies by 47%, but there was no significant effect on the annual outpatient costs at CHCs. Meanwhile, we did not observe the impact of the long-term prescription policy on patients’ annual number of hospitalisations.

**Conclusions:**

The long-term prescription policy mainly affected patients’ health care utilisation at CHCs and did facilitate patients with chronic diseases to refill drugs conveniently. The policy impact on patient health outcomes needs to be further observed and more attention should be given to the factors that may influence family doctors’ behaviour in delivering the long-term prescription service.

Due to their increasing global burden, the management of chronic diseases has become a public health priority globally [1]. Based on substantial evidence, this can best be done through the strengthening of primary health care [2]. In China, the prevalence of chronic disease has reached 34.3% [3], with hypertension being a major issue, affecting approximately 245 million individuals [4]. Although the country has made efforts to establish the family doctor system and place hypertensive patients as the key population for chronic disease management, control remains unsatisfactory at a 16% rate, which is below the global average of 21% [5]. This highlights the need for improved chronic disease management in primary care.

Internationally, long-term prescriptions account for most prescriptions in primary care, therein significantly influencing chronic disease management [6]. Many high-income countries have well-established long-term prescription policies, authorising family doctors or pharmacists to issue such prescriptions for patients with stable conditions requiring ongoing medication, which enhance the convenience of and access to medication [7–10]. In England, patients can obtain up to three-month prescription fills following a pharmacist’s review [8], while in the USA and Australia, the duration of long-term prescriptions ranges from one to two months [7].

Unlike many countries with long-term prescription policies, China has not established a gatekeeping system, as patients can voluntarily register with family doctors and seek treatment at higher-level hospitals without referrals [11]. Additionally, the short duration of prescriptions (typically less than one month) leads to patient frequent visits, which may decrease the continuity of primary care [12]. To enhance the role of primary care as the first point of contact and to satisfy patients’ long-term medication needs, some cities in China have implemented a long-term prescription policy since 2016. The policy supplements the family doctor system, allowing registered patients with stable chronic conditions to receive prescriptions for one to three months from family doctors in a single visit. The eligibility criteria for patients, the list of medications, and the duration of prescriptions vary across different pilot areas [13].

Previous studies have predominantly examined the impact of long-term prescription policy on health care utilisation and costs in high-income countries. Evidence from the USA has indicated that such a policy improved the medication adherence among patients with hypertension [14], diabetes [15], and asthma [16], and reduced drug costs [17,18]. Research in China have evaluated the impact of introducing the long-term prescription policy based on the family doctor system, indicating that it can reduce the total number of annual visits [19–21] and increase the proportion of visits at community health centres (CHCs) [22]. Studies have also shown that the policy significantly reduced annual health care costs [19,23], but also increased health care costs at CHCs specifically [24]. However, few studies to date have focussed on the net impact of the long-term prescription policy on primary care utilisation and costs, and have rarely provided empirical evidence based on longitudinal data.

In this study, we aimed to evaluate the net impact of the long-term prescription policy on primary care utilisation and costs among hypertensive patients using six-year longitudinal data. Our findings could inform strategies targeted at improving or promoting the long-term prescription policy and chronic disease management in primary care in China.

## METHODS

### Family doctor system and long-term prescription policy in City A

We conducted this study in a provincial capital city in eastern China, referred to as City A for the purpose of anonymising individual participant data. In 2019, City A had a resident population of 10.36 million, and the *per capita* disposable income was USD 8490.11.

The family doctor system in City A was initiated in 2015 in order to improve health management for residents, particularly for those with hypertension and diabetes. Residents can voluntarily choose a family doctor in a nearby CHC to register, with registration dates contingent upon their preferences. Besides general diagnosis and treatment services, registered residents receive personalised health management and consultation, quick referrals, and annual health assessments from family doctors.

To further improve the family doctor system, all the CHCs in City A have implemented the long-term prescription policy since 2018. The target population were patients who have been registered with a family doctor for over six months, those who have been diagnosed with 16 chronic conditions (*i.e.* hypertension, diabetes, Alzheimer disease, Parkinson disease, coronary heart disease, chronic renal disease), and those who have stable chronic condition and require long-term medication. Family doctors are the prescribers of long-term prescriptions. They are responsible for evaluating patient health status, informing patients of considerations for long-term prescriptions, and ensuring that any prescription is safe and appropriate. Prior to the implementation of the policy, patients receive supplies of medicine for up to two weeks or one month in a single visit, depending on the specific chronic disease. The policy further allows eligible residents to obtain up to three-month prescriptions (only for 16 chronic diseases) from family doctors in a single visit based on their chronic conditions, marking a substantial extension from the previous duration of prescriptions. Regarding the medication refill mechanism, patients are allowed to apply for refills when the remaining drug dosage of the prescription is less than seven days. Compared to other pilot cities in China, the long-term prescription policy in City A includes more chronic conditions and permits longer duration of prescriptions [13].

### Data source

There were three sources of data for this study. Within the health management information database, we identified all patients diagnosed with hypertension before 2015 and extracted their social demographics and chronic disease management information (such as blood pressure values at each follow-up visit) in 2014. The family doctor registration database provided information on residents’ register status from 1 January 2015 to 31 December 2019, including register organisation and dates. The medical insurance claims database recorded patients’ utilisation and costs of inpatient and outpatient care from 1 January 2014 to 31 December 2019. These three databases were linked via patients’ unique identification codes.

### Study population

We included individuals enrolled in City A’s municipal medical insurance, diagnosed with hypertension before 2015 and included in chronic disease management, visiting CHCs annually from 2014 to 2019, and either registered with a family doctor between 2015 and 2018 and remained registered until 2019 or not registered at all from 2015 to 2019. We then extracted the included patients’ records of health care utilisation and costs to compile a six-year longitudinal cohort data set.

### Outcomes

We investigated a variety of outcomes related to health care utilisation and costs in primary care. The indicators of health care utilisation were the average annual number of outpatient visits at CHCs, the average annual number of outpatient visits at pharmacies, the interval of prescriptions at CHCs, and the interval of prescriptions at pharmacies. Here, the interval of prescriptions refers to the number of days between consecutive visits in which patients incurred drug costs at CHCs or pharmacies. The indicators of health costs, meanwhile, were the average annual outpatient costs at CHCs and the average annual drug costs at pharmacies. Moreover, we used the average annual number of hospitalisations as an indicator to measure patient health outcomes.

### Covariates

We selected several covariates to control for the sociodemographic characteristics, such as age, sex, and type of medical insurance. The lattermost of these served as a proxy for the patient’s socioeconomic status, with patients with Urban Employee Basic Medical Insurance considered as having a higher socioeconomic status than those with Urban and Rural Resident Basic Medical Insurance. We also included body mass index (BMI) status, systolic blood pressure scores, and diastolic blood pressure scores to account for patients’ health conditions, and we calculated the number of comorbidities to further control for the severity of hypertension. This included common hypertension comorbidities such as diabetes, cerebrovascular disease, cardiovascular disease, and nephropathic disease, alongside the raw number of disease categories that the patient had (Table S1 in the **Online Supplementary Document**).

### Statistical analysis

We aimed to evaluate the net impact of the long-term prescription policy. In our study setting, residents registered gradually since 2015 and could be influenced by the family doctor system in four time periods: 2015–19, 2016–19, 2017–19, or 2018–19. The long-term prescription policy has been implemented as a supplement to the family doctor system in 2018. This meant that registered residents had been concurrently influenced by these two policies in 2018–19. Non-registered residents were not affected by either policy. To address this complexity, we chose the multi-stage difference-in-differences (DID) analysis, appropriate for interventions adopted at different times. In doing so, we followed the methodology of Mason et al. [25] and applied a multi-stage DID model incorporating two binary policy variables to accurately examine the policy’s net impact.

We used the following rules to determine intervention groups (**Table 1**). To evaluate the impact of the family doctor system, the treatment group consisted of patients who registered with family doctors between 2015 and 2018, while the control group comprised those individuals who were not registered. Meanwhile, we evaluated the impact of the long-term prescription policy by taking the patients who were eligible for the policy (registered with family doctors) as the treatment group and those who were not eligible (non-registered) as the control group. The sample population was categorised into five groups based on their registration status and timing. We provided descriptive statistics for baseline characteristics, primary care utilisation, costs, and hospitalisations among each group of hypertensive patients. Primary care costs from 2014 to 2019 were transformed to 2019’s price using the consumer price index, and all costs were reported in USD using the exchange rate of 2019 (USD 1 = CNY 6.98 in 2019).

**Table 1:** Groups of the study population*

Group	2014	2015	2016	2017	2018	2019
Non-registered	NI	NI	NI	NI	NI	NI
2015-registered	NI	R	R	R	RI	RI
2016-registered	NI	NI	R	R	RI	RI
2017-registered	NI	NI	NI	R	RI	RI
2018-registered	NI	NI	NI	NI	RI	RI

NI – not influenced by the policy in this year, R – registered with family doctors in this year, RI – registered with family doctors and influenced by the policy in this year

The expression of the multi-stage DID model was as follows:



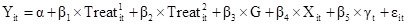



The outcome variable *Y_i_^t^* represents the primary care utilisation, costs, and hospitalisations of sample patient *i* in year *t* (from 2014 to 2019). *Treat_i_t^1^ r*epresents whether patient *i* was registered with a family doctor in year *t*. The intervention point was the first year of registration, and the dummy variable took the value of 0 before and 1 after the intervention point. *Treat_i_t^2^* represents whether patient *i* was influenced by the long-term prescription policy in year *t*. If patient *i* was eligible for the policy (*i.e.* registered in 2018 or 2019), then the value was to be 1; otherwise, it was 0. In this expression, we used *Treat_i_t^1^* and *Treat_i_t^2^* to denote the interaction of group and time in the traditional DID model. *G* included different types of family doctor system participants. *X_i_t* is a set of control variables, which capture patient *i*’s sociodemographic and health conditions in year *t*. *γt* is a set of time period dummy variables and *ε_i_t* is the random error term. *β_1_* refers to the average treatment effect of the family doctor system. *β_2_* is the estimated coefficient of interest, which reveals the average treatment effect of the long-term prescription policy affecting primary care utilisation, costs, and hospitalisations (Appendix S1 in the **Online Supplementary Document****)**.

For all analyses, we used Stata, version 17.0 (StataCorp, College Station, Texas, US).

## RESULTS

### Characteristics of the study population

A total of 164 857 hypertensive patients were included in the analysis (**Table 2**). There were 103 475 registered hypertensive patients in 2015, 33 089 in 2016, 13 150 in 2017, and 5544 in 2018, with an additional 9599 patients not registered overall. At baseline, the average age of the patients was 69.93 years. They were predominantly female (53.68%) and covered by Urban Employees Basic Medical Insurance (85.57%). Almost half (44.54%) had a BMI within the normal range. The average systolic blood pressure was 0.94 and diastolic was 0.98. Almost one-fourth (24.45%) of hypertensive patients had other comorbidities, with diabetes being the most common, affecting 22.17% of the population.

**Table 2:** Baseline characteristics of hypertensive patients in 2014*

	Total	Non-registered	Registered in 2015	Registered in 2016	Registered in 2017	Registered in 2018
**Demographic characteristics**						
Number of people	164 857	9599	103 475	33 089	13 150	5544
Age in year, x̄ (SD)	69.93 (11.18)	68.26 (12.57)	71.12 (10.74)	68.15 (11.46)	66.96 (11.44)	68.38 (11.22)
Age in years						
*<50*	6453 (3.91)	767 (7.99)	2583 (2.50)	1907 (5.76)	886 (6.74)	310 (5.59)
*50–60*	26 803 (16.26)	1866 (19.44)	14 391 (13.91)	6571 (19.86)	3001 (22.82)	974 (17.57)
*60–70*	53 935 (32.72)	2827 (29.45)	33 474 (32.35)	11 231 (33.94)	4435 (33.73)	1968 (35.50)
*70–80*	46 508 (28.21)	2383 (24.83)	31 467 (30.41)	8141 (24.60)	3085 (23.46)	1432 (25.83)
*>80*	31 158 (18.90)	1756 (18.29)	21 560 (20.84)	5239 (15.83)	1743 (13.25)	860 (15.51)
Sex						
*Female*	88 497 (53.68)	4632 (48.26)	57 990 (56.04)	16 729 (50.56)	6441 (48.98)	2705 (48.79)
*Male*	76 360 (46.32)	4967 (51.74)	45 485 (43.96)	16 360 (49.44)	6709 (51.02)	2839 (51.21)
**Type of medical insurance**						
Resident’s medical insurance	23 796 (14.43)	135 (1.41)	20 993 (20.29)	2154 (6.51)	404 (3.07)	110 (1.98)
Employee’s medical insurance	141 061 (85.57)	9464 (98.59)	82 482 (79.71)	30 935 (93.49)	12 746 (96.93)	5434 (98.02)
**BMI status**						
Normal	73 435 (44.54)	4985 (51.93)	45 120 (43.60)	14 777 (44.66)	6051 (46.02)	2502 (45.13)
Below normal	3701 (2.24)	248 (2.58)	2413 (2.33)	683 (2.06)	238 (1.81)	119 (2.15)
Above normal	87 721 (53.21)	4366 (45.48)	55 942 (54.06)	17 629 (53.28)	6861 (52.17)	2923 (52.72)
**Systolic blood pressure scores, x̄ (SD)**	0.94 (0.12)	0.94 (0.14)	0.95 (0.11)	0.94 (0.12)	0.93 (0.15)	0.93 (0.15)
**Diastolic blood pressure scores, x̄ (SD)**	0.98 (0.07)	0.98 (0.08)	0.98 (0.06)	0.98 (0.08)	0.97 (0.10)	0.97 (0.10)
**Comorbidities**						
0	124 546 (75.55)	7910 (82.40)	76 477 (73.91)	25 287 (76.42)	10 526 (80.04)	4346 (78.39)
1	20 057 (12.17)	502 (5.23)	13 747 (13.29)	3984 (12.04)	1296 (9.86)	528 (9.52)
2	20 144 (12.22)	1185 (12.35)	13 168 (12.73)	3801 (11.49)	1323 (10.06)	667 (12.03)
≥3	110 (0.06)	2 (0.02)	83 (0.08)	17 (0.05)	5 (0.04)	3 (0.05)
**Diabetes mellitus**	36 544 (22.17)	1471 (15.32)	24 437 (23.62)	7172 (21.67)	2397 (18.23)	1067 (19.25)

SD – standard deviation, x̄ – mean

*All values are expressed as number (%) unless specified otherwise.

### Primary care utilisation

We observed an increase in primary care utilisation among hypertensive patients since the initiation of the family doctor system in 2015. The average annual number of outpatient visits at CHCs rose from 15.51 in 2014 to 19.82 in 2017, and the interval of prescriptions at CHCs was shortened from 30.08 days to 24.50 days (**Table 3**). Concurrently, the average annual number of outpatient visits at pharmacies decreased.

**Table 3:** Hypertensive patients’ primary care utilisation in 2014–19, presented as mean (standard deviation)

	Total	Non-registered	Registered in 2015	Registered in 2016	Registered in 2017	Registered in 2018
**Average annual number of outpatient visits at CHCs (days)**						
2014	15.51 (11.66)	5.66 (8.36)	17.77 (11.61)	14.25 (10.94)	10.68 (9.85)	9.39 (9.92)
2017	19.82 (12.96)	5.29 (8.21)	22.03 (13.01)	19.53 (11.91)	16.53 (10.22)	13.32 (10.12)
2019	16.10 (11.69)	5.30 (7.41)	17.62 (11.83)	15.79 (11.25)	13.96 (10.06)	13.29 (9.92)
**Average annual number of outpatient visits at pharmacies**						
2014	4.82 (7.60)	5.74 (8.60)	4.32 (7.11)	5.52 (8.23)	5.72 (8.19)	6.30 (8.56)
2017	3.48 (7.30)	6.22 (9.83)	2.81 (6.44)	3.86 (7.82)	4.63 (8.13)	6.26 (9.44)
2019	2.56 (5.45)	5.15 (7.57)	2.10 (4.88)	2.81 (5.63)	3.21 (6.15)	3.77 (6.59)
**Interval of prescriptions at CHCs**						
2014	30.08 (31.88)	44.53 (50.19)	27.68 (27.74)	31.76 (33.32)	37.75 (42.03)	40.30 (45.34)
2017	24.50 (20.58)	43.68 (47.92)	22.79 (17.10)	24.81 (19.05)	27.65 (22.95)	32.50 (33.30)
2019	29.72 (26.73)	44.31 (47.88)	27.81 (24.30)	31.06 (26.75)	34.01 (29.40)	34.66 (30.49)
**Interval of prescriptions at pharmacies**						
2014	48.31 (54.39)	42.69 (48.60)	50.01 (55.72)	47.10 (53.71)	45.35 (51.72)	44.46 (50.51)
2017	53.70 (60.76)	46.54 (52.93)	55.30 (62.53)	54.40 (60.90)	51.60 (57.82)	48.81 (56.69)
2019	54.85 (59.35)	48.11 (52.95)	55.78 (60.40)	55.69 (59.87)	55.21 (59.05)	52.26 (55.75)

CHC – community health centres

With the implementation of the long-term prescription policy, the average annual number of outpatient visits at CHCs was 16.10 in 2019, with a decrease of 18.77% compared to 2017. The annual number of outpatient visits at pharmacies continued to decline, from 3.48 in 2017 to 2.56 in 2019. The interval of prescriptions at CHCs was 29.72 days, a 21.31% extension compared to 2017. The interval of prescriptions at pharmacies also extended, from 53.70 days in 2017 to 54.85 days in 2019. Further comparison between registered and non-registered groups showed a decrease in the average annual number of outpatient visits at CHCs across all registered groups, whereas the non-registered group exhibited an increasing trend.

### Primary care costs

The average annual outpatient costs at CHCs among hypertensive patients were USD 1006.74 in 2017, reflecting a growth rate of 49.36% from 2014 (**Table 4**). The average annual drug costs at pharmacies also increased from USD 128.46 in 2014 to USD 133.23 in 2017.

**Table 4:** Hypertensive patients’ primary care costs in 2014–19, presented as mean (standard deviation)

	Total	Non-registered	Registered in 2015	Registered in 2016	Registered in 2017	Registered in 2018
**Average annual outpatient costs at CHCs**						
2014	674.03 (1017.71)	176.00 (510.23)	548.80 (621.90)	442.15 (575.78)	313.31 (507.24)	289.08 (583.49)
2017	1006.74 (1387.10)	187.47 (456.86)	830.97 (797.65)	744.22 (756.22)	600.13 (634.25)	481.73 (596.89)
2019	990.48 (1282.90)	207.19 (414.55)	828.21 (818.81)	771.44 (799.41)	662.43 (697.01)	636.74 (689.27)
**Average annual drug costs at pharmacies**						
2014	128.46 (338.34)	168.19 (429.60)	110.36 (298.22)	152.34 (381.89)	160.37 (395.74)	179.27 (406.86)
2017	133.23 (497.05)	254.57 (703.07)	105.05 (432.09)	151.20 (540.66)	175.03 (570.54)	242.56 (656.74)
2019	95.97 (502.75)	201.06 (674.25)	77.46 (438.82)	106.52 (572.72)	117.01 (560.06)	146.63 (635.22)

CHC – community health centres

The average annual outpatient costs at CHCs were USD 990.48 in 2019, showing a decrease of 1.62% from 2017, meaning after the implementation of the long-term prescription policy. Costs at CHCs among patients in the non-registered group were relatively low, but exhibited an upward trend from 2017 to 2019. The trend of costs at CHCs varied among the registered groups, with the 2015-registered group experiencing a decline and other three groups seeing an increase.

In 2019, the average annual drug costs at pharmacies among hypertensive patients was USD 95.97, marking a decrease of 27.96% from 2017. Costs between 2017 and 2019 were relatively high for patients in the non-registered group and relatively low for those in the earlier registered group. However, costs in all groups declined to varying degrees.

### Health outcomes: hospitalisations

The average annual number of hospitalisations among hypertensive patients increased each year, from 0.20 in 2014 to 0.45 in 2019 (**Table 5**). Hospitalisations and their growth rates were higher in the non-registered group than in all other registered groups of patients.

**Table 5:** Hypertensive patients’ hospitalisations in 2014–19, presented as mean (standard deviation)

Group	Total	Non-registered	Registered in 2015	Registered in 2016	Registered in 2017	Registered in 2018
2014	0.20(0.63)	0.28(0.95)	0.19(0.61)	0.18(0.58)	0.19(0.59)	0.21(0.67)
2017	0.28(0.86)	0.38(1.31)	0.28(0.83)	0.25(0.79)	0.24(0.80)	0.31(0.97)
2019	0.45(1.37)	0.60(1.89)	0.45(1.34)	0.41(1.32)	0.40(1.29)	0.44(1.38)

### The net impact of long-term prescription policy: regression results

We estimated the net impact of the long-term prescription policy on primary care utilisation, costs, and hospitalisations using a multi-stage DID analysis (**Table 6**). The regression results showed that the long-term prescription policy significantly reduced the average annual outpatient visits by 2.47 at CHCs (*P* < 0.001) and 0.18 at pharmacies (*P* = 0.018) (Table S2–4 in the **Online Supplementary Document****)**. It also significantly prolonged the interval of prescriptions by 3.10 days at CHCs (*P* < 0.001), while having no effect on the interval of prescriptions at pharmacies (*P* = 0.974). In terms of costs, the average annual drug costs at pharmacies decreased by 47% (*P* < 0.001), but there was no significant change in the average annual outpatient costs at CHCs (*P* = 0.240). The impact on the average annual number of hospitalisations, meanwhile, was not statistically significant (*P* = 0.147).

**Table 6:** The net impact of the long-term prescription policy on primary care utilisation, costs, and hospitalisations among hypertensive patients

Variables	DID estimate (SE)	*P*-value	Observations	R^2^
Average annual number of outpatient visits at CHCs	−2.47 (−32.80)	<0.001	989 142	0.141
Average annual number of outpatient visits at pharmacies	−0.18 (−2.37)	0.018	989 142	0.051
Interval of prescriptions at CHCs	3.10 (5.18)	<0.001	930 106	0.042
Interval of prescriptions at pharmacies	−0.02 (−0.03)	0.974	432 027	0.006
Average annual outpatient costs at CHCs	0.04 (1.18)	0.240	989 142	0.316
Average annual drug costs at pharmacies	−0.47 (−15.70)	<0.001	989 142	0.105
Average annual number of hospitalisations	0.04 (1.45)	0.147	989 142	0.044

CHC – community health centre, DID – difference-in-differences, SE – standard error, R^2^ – coefficient of determination

## DISCUSSION

To our knowledge, this is the first study to examine the net impact of the long-term prescription policy on primary care utilisation and costs in China using longitudinal data. The findings indicate that the policy was associated with a significant decrease in outpatient visits for hypertension patients at both CHCs and pharmacies, as well as an extension of the prescription intervals at CHCs. Regarding health care costs, the policy significantly reduced drug costs at pharmacies but had no significant impact on outpatient costs at CHCs. It did not, however, significantly influence patient hospitalisation rates. In summary, the long-term prescription policy mainly affected health care utilisation at CHCs.

The long-term prescription policy in China aims to provide convenient drug refills for patients with chronic diseases, address their long-term medication needs, and promote a hierarchical system of diagnosis and treatment. We found that the policy increased prescription intervals by an average of 3.10 days and reduced the annual number of outpatient visits to CHCs by 2.47 among hypertensive patients, suggesting that it effectively minimised patients’ repeated visits to CHCs for simple drug refills. Our results differ from a previous study that reported an increase in CHC visits due to the policy [24]. However, we used a multi-stage DID model which included non-registered residents as a comparison group, analysed a larger sample size, and evaluated the policy’s impact over two years post-implementation. These factors likely contributed to the greater accuracy of our findings.

The long-term prescription policy had only a limited impact on extending prescription intervals. We speculate that certain factors may influence family doctors’ willingness to provide long-term prescription services. Previous studies have highlighted concerns among family doctors regarding drug safety, the limited drug lists for long-term prescriptions, and potential income loss from fewer patient visits [13,26–28]. To address these issues, it is crucial to foster a better understanding of the long-term prescription policy among family doctors, monitor the medication outcomes of patients using the service, expand the range of eligible diseases and drugs, and implement appropriate incentive mechanisms for family doctors.

We further found that the long-term prescription policy significantly reduced pharmacy costs. This reduction was attributed to registered patients filling their prescriptions at CHCs instead of pharmacies, leading to decreased pharmacy utilisation. The policy also had no significant impact on outpatient costs at CHCs, as its primary goal was to extend prescription durations rather than reduce the overall volume of medication used by patients.

We did not observe an impact of the long-term prescription policy on patient hospitalisations. While hypertension itself rarely causes hospitalisations, its complications can increase hospitalisation risks [29]. Our findings suggest that, despite fewer health care visits following the policy’s implementation, patients maintained effective disease control, indicating their conditions did not worsen. This finding has important implications for controlling the rising hospitalisation rates in China [30].

This study highlighted the contrasting effects of the family doctor system and the long-term prescription policy on health care utilisation among hypertensive patients, as shown by the multi-stage DID model. Consistent with previous studies [31,32], we found that the family doctor system increased patient visits at CHCs. This system reduces reliance on secondary and tertiary health care by encouraging chronic disease patients to seek care at CHCs. In contrast, our findings showed that the long-term prescription policy reduced patient visits to CHCs. Some studies suggest that patients using long-term prescription services develop closer relationships with their family doctor, enabling better chronic disease management and reducing unnecessary visits [22]. These findings indicate that the long-term prescription policy complements the family doctor system by improving its efficiency and performance. Together, they strengthen primary care’s role as the first point of contact in the health care system.

Several limitations must be acknowledged. First, we evaluated the impact of the long-term prescription policy only within two years of its implementation. This timeframe may be insufficient to fully capture its effects on patient health outcomes. Second, while we controlled for major confounders, differences remained between the treatment and the control groups. We also did not collect certain potential confounding variables, such as education levels, access to health care, and health-seeking behaviours. Future studies should incorporate more comprehensive data to provide a more accurate assessment of the policy’s effects. Third, we conducted this study in a pilot city which has a population of a higher economic status and which adopted the family doctor system earlier than other cities in China. While our findings are generalisable to regions with more advanced family doctor systems, they may not apply to rural or less developed areas with lower family doctor registration rates. Future studies could examine the policy’s effects as the family doctor system expands in these regions. Lastly, we did not account for variations in practices among health care providers. Future research could focus on the effects of extending different prescription durations on patient health care utilisation and health outcomes.

## CONCLUSIONS

In this study, we examined the net impact of the long-term prescription policy on primary care utilisation and costs. The results showed that the policy is associated with a significant extension of prescription intervals and reduced outpatient visits at CHCs, demonstrating its effectiveness in providing patients with chronic conditions a convenient way to refill drugs. However, further attention is needed to address factors that may influence family doctors’ delivery of long-term prescription services. Additionally, ongoing observation is essential to monitor the policy’s impact over time. Future studies should aim to collect longer-term and more detailed data and to comprehensively assess the policy’s effects on patient health outcomes, including medication adherence, chronic disease management, and the development of new complications. Exploring the policy’s effect on other chronic diseases could provide additional valuable insights.

## Additional material


Online Supplementary Document
