# Supplementary material for: The impact of long-term prescription policy on primary care utilisation and costs among hypertensive patients in China: a six-year longitudinal study
Source: J Glob Health. 2025 Jan 17;15:04021. doi: 10.7189/jogh.15.04021 (PMC11737817; doi:10.7189/jogh.15.04021)
Supplement: Online Supplementary Document [file jogh-15-04021-s001.pdf]

Supplement to:Yu C, Zhang L, Zhang L, Chen W. The impact of long-term prescription policy on primary care utilisation and costs among hypertensive patients in China: a six-year longitudinal study. J Glob Health. 2025;15:04021.

Table S1. Control variables in multi-stage DID model

Variable Name	Description
Age (years)	Categorical variable:18-50; 51-60; 61-70; 71-80; >81
Sex	Binary variable: male; female
Register group	Categorical variable: non-registered group; 2015-registered group; 2016-registered group; 2017-registered group; 2018-registered group
Type of medical insurance	Binary variable: Urban Employee Basic Medical Insurance; Urban and rural Resident Basic Medical Insurance
BMI type (kg/m^2)	Categorical variable: normal: 18.5~24; below normal: <18.5; above normal: >24
Systolic blood pressure scores	Continuous variable: 0~1, the closer to 1, the better. When patients' systolic blood pressure values in follow-up visits were normal (90mmHg~139mmHg), the scores were taken as 1, otherwise they were taken as 0. Then scores were averaged for multiple follow-up visits in 2014.
Diastolic blood pressure scores	Continuous variable: 0~1, the closer to 1, the better. When patients' diastolic blood pressure values in follow-up visits were normal (60mmHg~89 mmHg), the scores were taken as 1, otherwise they were taken as 0. Then scores were averaged for multiple follow-up visits in 2014.
Comorbidities	Categorical variable: 0, 1, 2, ⩾3

Hypertension Comorbidities:

(1) Diabetes: E10-E14.

(2) Cerebrovascular diseases: cerebral hemorrhage (I61); cerebral infarction (I66, I69); stroke (I63, I64); cerebral atherosclerosis; cerebrovascular disease; cerebral blood supply insufficiency (I67, R42).

(3) Cardiovascular diseases: atrial and ventricular enlargement; hypertrophy; and atrial-ventricular thrombosis (I51); coronary artery disease (I24, I25, I70); cardiac arrhythmia (I49); hypertensive heart disease (I11); hypertensive heart failure (I11, I25, I50); angina pectoris (I20); myocardial infarction (I21, I22); aneurysm of aortic coarctation (I71); atrial fibrillation (I48), and ischemia of the posterior circulation (G45.004).

(4) Nephropathic disease: hypertensive nephropathy (I12); hypertensive heart and kidney disease with heart failure or renal failure (I13).

(5) Hypertensive hemiparesis: G81.9.

Full regression results of multi-stage difference-in-differences

Table S2. The impact of family doctor system and the long-term prescription policy on primary care utilization among patients with hypertension

Variables	Average annual number of outpatient visits at CHCs	Average annual number of outpatient visits at pharmacies	Interval of prescriptions at CHCs	Interval of prescriptions at pharmacies
Family doctor system	2.95**	-1.10**	-2.25**	2.16**
	(58.92)	(-30.04)	(-14.97)	(5.96)
Long-term prescription policy	-2.47**	-0.18*	3.10**	-0.02
	(-32.80)	(-2.37)	(5.18)	(-0.03)
Register Group (control: non-registered)				
2015-registered	12.13**	-1.35**	-16.93**	6.00**
	(207.30)	(-24.98)	(-44.46)	(13.33)
2016-registered	10.29**	-0.78**	-15.03**	4.80**
	(180.42)	(-14.43)	(-39.77)	(11.03)
2017-registered	7.55**	-0.41**	-11.07**	3.34**
	(127.86)	(-7.23)	(-28.61)	(7.29)
2018-registered	5.76**	0.32**	-8.06**	0.77
	(81.14)	(4.70)	(-18.85)	(1.47)
Year (control: 2014)				
2015	1.09**	-0.06	-1.42**	0.10
	(22.61)	(-1.63)	(-8.80)	(0.31)
2016	1.30**	-0.49**	-3.53**	4.07**
	(23.55)	(-11.93)	(-19.78)	(10.15)
2017	1.90**	-0.38**	-3.94**	3.58**
	(31.78)	(-8.87)	(-21.39)	(8.29)
2018	0.86**	-0.78**	-5.66**	0.72
	(10.86)	(-9.85)	(-9.29)	(1.08)
2019	0.59**	-1.12**	-1.99**	4.58**
	(7.33)	(-14.28)	(-3.26)	(6.83)
Sex (control: female)	-0.76**	-0.09**	0.64**	0.03
	(-31.76)	(-6.33)	(12.09)	(0.19)
Age (control: <50)				
50~60	0.88**	-0.67**	-1.42**	1.73**
	(19.02)	(-19.01)	(-10.45)	(4.14)
60~70	2.20**	-0.96**	-3.05**	1.14**
	(43.78)	(-27.25)	(-22.29)	(2.59)
70~80	3.37**	-0.93**	-4.72**	0.39
	(64.02)	(-25.79)	(-33.89)	(0.86)
>80	3.59**	-0.79**	-5.31**	-0.34
	(60.67)	(-20.38)	(-35.74)	(-0.71)

(continued) Table S2. The impact of family doctor system and the long-term prescription policy on primary care utilization among patients with hypertension

Variables	Average annual number of outpatient visits at CHCs	Average annual number of outpatient visits at pharmacies	Interval of prescriptions at CHCs	Interval of prescriptions at pharmacies
Type of medical insurance (control: resident’ s medical insurance)				
Employee’ s medical	0.84**	1.15**	-0.91**	-0.86*
insurance	(20.75)	(44.15)	(-8.95)	(-2.32)
Systolic blood pressure scores	-0.03	-0.29**	0.50	1.34
	(-0.31)	(-4.16)	(1.75)	(1.61)
Diastolic blood pressure scores	0.16	-1.06**	0.83	4.42**
	(0.95)	(-8.13)	(1.57)	(3.16)
BMI status (control: normal)				
Below normal	0.20*	0.07	-0.61**	-1.09*
	(2.40)	(1.57)	(-3.46)	(-1.86)
Above normal	0.30**	-0.03	-0.67**	0.21
	(12.94)	(-1.91)	(-12.72)	(1.20)
Comorbidities (control: 0)				
1	3.34**	0.33**	-3.95**	-3.11**
	(86.29)	(15.24)	(-57.13)	(-11.32)
2	3.26**	0.42**	-3.90**	-4.10**
	(83.47)	(18.29)	(-54.14)	(-15.49)
⩾3	6.67**	0.65*	-8.46**	-2.15
	(12.12)	(2.29)	(-18.47)	(-0.60)
Constant term	1.12**	7.35**	49.58**	38.16**
	(7.14)	(57.21)	(82.39)	(29.38)
Sample size	989,142	989,142	930,106	432,027
R-squared	0.141	0.051	0.042	0.006

**P* <0.05, ***P* <0.01

Table S3. The impact of family doctor system and the long-term prescription policy on primary care costs among patients with hypertension

Variables	Average annual outpatient costs at CHCs	Average annual drug costs at pharmacies
Family doctor system	0.42**	-0.58**
	(47.04)	(-39.79)
Long-term prescription policy	0.04	-0.47**
	(1.18)	(-15.70)
Register Group (control: non-registered)		
2015-registered	3.61**	-0.42**
	(191.77)	(-20.64)
2016-registered	3.46**	-0.08**
	(186.05)	(-3.94)
2017-registered	3.01**	0.16**
	(155.69)	(7.70)
2018-registered	2.48**	0.37**
	(111.02)	(15.05)
Year (control: 2014)		
2015	0.10**	-0.09**
	(10.33)	(-6.37)
2016	0.25**	-0.32**
	(23.00)	(-19.80)
2017	0.40**	-0.24**
	(35.02)	(-13.82)
2018	0.27**	0.02
	(8.33)	(0.66)
2019	0.35**	-0.16**
	(10.98)	(-5.10)
Sex (control: female)	-0.06**	-0.07**
	(-18.87)	(-11.14)
Age (control: <50 years old)		
50~60	0.12**	-0.46**
	(15.17)	(-30.55)
60~70	0.21**	-0.67**
	(26.71)	(-43.38)
70~80	0.30**	-0.69**
	(37.13)	(-43.86)
>80	0.30**	-0.65**
	(33.82)	(-38.39)
Type of medical insurance (control: resident's medical insurance)		
Employee's medical insurance	0.16**	0.87**
	(28.23)	(73.59)

(continued) Table S3. The impact of family doctor system and the long-term prescription policy on primary care costs among patients with hypertension

Variables	Average annual outpatient costs at CHCs	Average annual drug costs at pharmacies
Systolic blood pressure scores	-0.09**	-0.09**
	(-5.29)	(-2.92)
Diastolic blood pressure scores	0.21**	-0.37**
	(6.76)	(-7.00)
BMI status (control: normal)		
Below normal	-0.06**	-0.01
	(-5.56)	(-0.40)
Above normal	0.11**	0.00
	(34.56)	(0.32)
Comorbidities (control: 0)		
1	0.54**	0.04**
	(122.72)	(4.39)
2	0.57**	0.06**
	(116.62)	(6.12)
⩾3	0.98**	0.27*
	(16.60)	(2.09)
Constant term	3.23**	4.69**
	(99.69)	(93.23)
Sample size	989,142	989,142
R-squared	0.316	0.105

*P <0.05, **P <0.01

Table S4. The impact of family doctor system and the long-term prescription policy on hospitalizations with hypertension

Variables	Average annual number of hospitalizations
Family doctor system	-0.02
	(-0.99)
Long-term prescription policy	0.04
	(1.45)
Register Group (control: non-registered)	
2015-registered	-0.31**
	(-14.66)
2016-registered	-0.34**
	(-16.40)
2017-registered	-0.29**
	(-13.21)
2018-registered	-0.21**
	(-8.49)
Year (control: 2014)	
2015	0.22**
	(14.67)
2016	0.31**
	(18.27)
2017	0.43**
	(24.05)
2018	0.62**
	(19.77)
2019	0.93**
	(29.78)
Sex (control: female)	0.10**
	(15.27)
Age (control: <50 years old)	
50~60	0.24**
	(12.86)
60~70	0.48**
	(24.80)
70~80	0.81**
	(42.29)
>80	1.09**
	(55.14)
Type of medical insurance	
(control: resident' s medical insurance)	
Employee' s medical insurance	0.04*
	(2.54)

(continued) Table S4. The impact of family doctor system and the long-term prescription policy on hospitalizations with hypertension

Variables	Average annual number of hospitalizations
Systolic blood pressure scores	-0.07*
	(-2.03)
Diastolic blood pressure scores	0.13*
	(2.16)
BMI status (control: normal)	
Below normal	0.17**
	(8.43)
Above normal	-0.02**
	(-2.78)
Comorbidities (control: 0)	
1	0.30**
	(33.22)
2	0.36**
	(40.26)
⩾3	1.00**
	(11.11)
Constant term	-2.25**
	(-40.44)
Sample size	989,142
R-squared	0.044

*P <0.05, **P <0.01

Appendix S1. Multi-stage DID model

This study aims to examine the impact of family doctor system and long-term prescription policy on primary care utilization, costs and hospitalizations. The expression of the multi-stage DID model was as follows:

$$Y_{it} = \alpha + \beta_1 * Treat_{it}^1 + \beta_2 * Treat_{it}^2 + \beta_3 * G + \beta_4 * X_{it} + \beta_5 * \gamma_t + \varepsilon_{it}$$

The outcome variable Y_{it} represents the primary care utilization, costs and hospitalizations of sample patient i in year t (from 2014 to 2019). $Treat_{it}^1$ represents whether patient i was registered with a family doctor in year t. The intervention point was the first year of registration, and the dummy variable took the value of 0 before and 1 after the intervention point. $Treat_{it}^2$ represents whether patient i was influenced by the long-term prescription policy in year t. If patient i was eligible for the policy (be registered in 2018 or 2019), then the value was to be 1; otherwise, it was 0. In this expression, we used $Treat_{it}^1$ and $Treat_{it}^2$ to denote the interaction of group and time in the traditional DID model. X_{it} is a set of control variables, which capture patient i's sociodemographic and health conditions in year t. G included different types of family doctor system participants (Table2). γ_t is a set of time period dummy variables. Its coefficients explain the effect of aggregate temporal factors that cause changes in the outcome. ε_{it} is the random error term. β_1 refers to the average treatment effect of the family doctor system. β_2 is the estimated coefficient of interest, which reveals the average treatment effect of the long-term prescription policy affecting primary care utilization, costs and hospitalizations. β_3 captures differences in rates with respect to the family doctor system reference group (never registered in family doctor system) prior to the intervention.

For instance, the difference in average outcomes between practices in the group 2015-registered and practices in the group non-registered in year 2014 after adjusting for practice characteristics is:

$$\overline{Y}_{t=2017}^{2015-registered} - \overline{Y}_{t=2017}^{non-registered} = (\alpha + \beta_{3,2015-registered} + \beta_{5,t=2017}) - (\alpha + \beta_{5,t=2017}) = \beta_{3,2015-registered}$$

The policy effects of the family doctor system and long-term prescription policy are captured by the coefficients β_1 and β_2.

For instance, the difference in outcomes for the group 2015-registered between 2014 and 2015 is:

$$\overline{Y}_{t=2015}^{2015-registered} - \overline{Y}_{t=2014}^{2015-registered} = (\alpha + \beta_1 + \beta_{3,2015-registered} + \beta_{5,t=2015}) - (\alpha + \beta_{3,2015-registered} + \beta_{5,t=2014}) = (\beta_{5,t=2015} - \beta_{5,t=2014}) + \beta_1$$

For the group non-registered between 2014 and 2015 is:

$$\overline{Y}_{t=2015}^{non-registered} - \overline{Y}_{t=2014}^{non-registered} = (\alpha + \beta_{3,2015-registered} + \beta_{5,t=2015}) - (\alpha + \beta_{5,t=2014}) = (\beta_{5,t=2015} - \beta_{5,t=2014})$$

Therefore, the difference-in differences estimate (which we call family doctor system variable) is:

$$(\overline{Y}_{t=2015}^{2015-registered} - \overline{Y}_{t=2014}^{2015-registered}) - (\overline{Y}_{t=2015}^{non-registered} - \overline{Y}_{t=2014}^{non-registered}) = \beta_1$$

Note, that β_3 appears in the calculation of the average outcome for the registered group in the post-intervention year 2015 $(\overline{Y}_{t=2015}^{2015-registered})$ but cancels out in DID estimate.

Similarly,

$$\beta_2 = ((\overline{Y}_{t=2018}^{2015-registered} - \overline{Y}_{t=2014}^{2015-registered}) - (\overline{Y}_{t=2018}^{non-registered} - \overline{Y}_{t=2014}^{non-registered})) - (\overline{Y}_{t=2015}^{2015-registered} - \overline{Y}_{t=2014}^{2015-registered}) - (\overline{Y}_{t=2015}^{non-registered} - \overline{Y}_{t=2014}^{non-registered})$$
